# Editorial

**Published:** 1855-07

**Authors:** 


### THE MEDICAL EXAMINER.

### PHILADELPHIA, JULY, 1855.

### PHILADELPHIA HOSPITAL, BLOCKLEY.

We learn, with much satisfaction, that the Board of Guardians of the Poor, of Philadelphia, have lately reorganized the Hospital Department of the Alms House, with a view to Clinical Instruction. A Medical Board has been appointed, consisting of "four surgeons and lecturers, on clinical surgery, four physicians and lecturers on clinical medicine and three accoucheurs and lecturers on clinical midwifery," and the following gentlemen have been elected to these posts :—

*Surgeons*—Drs. JOHN NEILL,
H. H. SMITH,
R. P. THOMAS,
D. H. AGNEW.

*Physicians*—Drs. JOHN B. BIDDLE,
J. L. LUDLOW,
JOSEPH CARSON,
H. HARTSHORNE.

*Accoucheurs*—Drs. WILSON JEWELL,
CASPAR MORRIS,
R. A. F. PENROSE.

The Philadelphia Alms House Hospital offers unequalled facilities for clinical instruction. Its inmates number between two and three thousand, and furnish the clinical wards with a material which in variety and interest is certainly unsurpassed in this country. The lecture room of the Hospital is spacious and commodious; and the short distance between it and the different medical schools, is now rapidly and conveniently travelled by rail-road cars.

We feel an especial gratification in these increased facilities which are thus opened for clinical instruction in Philadelphia, in the fact that they remove the only apology for those miserable substitutes for hospital instruction, the college clinics. Here, in Philadelphia at least, with the opportunities that are now within the reach of students, at the Pennsylvania Hospital and the Philadelphia Hospital, for the investigation of disease and pathology, there can be no longer any plea for diverting the medical classes from the only legitimate field for a practical knowledge of their profession.

