# Correction

**Published:** 1855-09

**Authors:** 


CORRECTION.

In the July number of the Examiner, our readers were informed that Dr. Caspar Morris was chosen one of the Accoucheurs to the Philadelphia Hospital, Blockley. It should have been printed, Consulting Physician to the Children's Department, and Lecturer on the Diseases of Children.

